# Risk factors for contracting watery diarrhoea in Mzilikazi, Bulawayo City, Zimbabwe, 2020: a case control study

**DOI:** 10.11604/pamj.2022.41.145.30551

**Published:** 2022-02-18

**Authors:** Tshebukani Mzingaye Moyo, Tsitsi Patience Juru, Edwin Sibanda, Gladys Marape, Notion Tafara Gombe, Emmanuel Govha, Mufuta Tshimanga

**Affiliations:** 1 Department of Primary Health Care Sciences, Family Medicine, Global and Public Health Unit, University of Zimbabwe, Harare, Zimbabwe,; 2 Department of Health, Bulawayo City Council, Bulawayo, Zimbabwe,; 3 African Field Epidemiology Network, Harare, Zimbabwe

**Keywords:** Diarrhoea, outbreak, Mzilikazi clinic, Bulawayo, Zimbabwe

## Abstract

**Introduction:**

Mzilikazi clinic had an upsurge of diarrhoea cases with 41 cases from the 28^th^ to the 30^th^ of September 2020, against a threshold of 11. We therefore, investigated the risk factors associated with this outbreak to recommend prevention and control measures.

**Methods:**

we conducted a 1:1 unmatched case-control study. A case was any person who suffered from diarrhoea, and was resident in the clinic´s catchment since the 21^st^ of September 2020. Demographic data, knowledge and practices related to diarrhoea were collected using a standard questionnaire for both cases and controls. Environmental assessment, water quality and stool testing was also done. We conducted univariate and multivariate analysis at 95% confidence interval, to determine factors independently associated with contracting diarrhoea.

**Results:**

the median age was 30 years (Q1=12, Q3=46) for cases and 30 years (Q1=22, Q3=48) for controls. The dominant gender was female for cases and male for controls. The independent risk factors were: drinking borehole water [adjusted Odds Ratio (aOR)=2.66; 95%CI=(1.41-5.00)], storing water in open container [aOR=2.76; 95%CI=(1.38-5.53)] and being under-five years old [aOR=5.73; 95%CI=(2.06-15.89)]. Boiling drinking water [aOR=0.39; 95%CI=(0.20-0.75)] was protective. Coliforms were detected from boreholes and stored water samples, and Shigella flexneri was isolated from 2 of the 13 stool specimens collected. Residents accessed water from decommissioned boreholes due to severe municipal water rationing.

**Conclusion:**

being under-five years old, drinking borehole water and storing water in open containers were independent risk factors. Health education on home water treatment, distribution of water storage containers, and Aquatabs was done.

## Introduction

Diarrhoea is defined as the passage of three or more loose or watery stools in 24 hours, and loose stool being one that takes shape of a container. Three clinical syndromes of diarrhoea can be identified namely acute watery diarrhoea, dysentery, and persistent diarrhoea [1]. Diarrhoea causes fluid, and electrolyte losses which if left unattended to, may cause death [2].

Diarrhoea is a common symptom of gastrointestinal infections that are caused by bacteria, protozoa, and viruses. Major bacterial pathogens that cause diarrhoea include *Escherichia Coli, Shigella, Vibrio Cholerae, Campylobacter*, and *Salmonella*. Viral etiological agents usually include *Rota virus, Corona*, and *Adenoviruses*. Diarrhoea due to parasitic or protozoal causes usually attributed to *Giardia, Entamoeba*, or *Cryptosporidium infection*. The mode of transmission of these pathogens is faeco-oral [3,4]. Annually, an estimated 2.5 billion cases of diarrhoea occur among the under five-year-old. More than half of these cases occur in South Asia, and Africa. Diarrhoeal diseases are the second leading cause of mortality among the under five-year-old children. Africa, and South Asia accounts for more than 80 percent of childhood mortality due to diarrhoea [3,5]. Approximately 88 percent of diarrhoea associated mortality is attributed to unsafe water, insufficient hygiene, and inadequate sanitation [6]. In light of this, WHO notes that access to safe water, improved sanitation, exclusive breast feeding for the first six months of infancy, good personal, and food hygiene are among the key measures to prevent diarrhoea [7]. On the 26^th^ of September 2020, the Bulawayo City health department received an alert from a community member that some residents were ill and complaining of diarrhoea and vomiting. That week (week 39), the health facility reported ten cases. Risk communication on diarrhoea prevention practices was initiated through door-to-door visits, social media platforms, and radio sessions. The following week (week 40), there was a spike in the cases reported by the clinic as 41 cases were reported from the 28^th^ to the 30^th^ of September 2020 surpassing the weekly action threshold of 11 cases. A watery diarrhoea outbreak was declared on the 30^th^ of September 2020. We therefore, investigated the watery diarrhoeal outbreak to identify the risk factors for contracting the disease.

## Methods

**Study design and population:** we conducted a 1:1 unmatched case-control study among residents of the Mzilikazi clinic catchment area. A case was defined as any person who presented with watery diarrhoea, with or without any other symptoms from the 28^th^ of September 2020, and was a resident in Mzilikazi clinic catchment area for one week prior to the onset of symptoms. A control was defined as any person who did not develop diarrhoea from the 28^th^ of September 2020 up to the day of the interview, and was a resident in Mzilikazi clinic catchment area for one week prior to the start of the outbreak.

**Study setting:** Mzilikazi clinic is located in the Northern suburbs district, and caters for the residents of ward seven and eight. The health facility has a catchment population of 45,425 and is located approximately six kilometers from Bulawayo central business district. Residents of the area have access to piped water. However, due to the decrease in the water levels at the dams servicing Bulawayo city, a water rationing program was instituted. Over time, the number of days without water increased with areas experiencing 144 hours a week without running tap water.

**Sample size:** using Stat Calc function of Epi InfoTM 7, assuming using untreated water is a significant risk factor for contracting diarrhoea with an odds ratio of 2.4, 30% exposure in controls and 50% exposure in cases (study by Maponga *et al*. risk factors for contracting watery diarrhoea in Kadoma City, Zimbabwe: a case-control study) using a power of 80%, confidence interval of 95%, and 10% refusal rate the calculated sample size was 106 cases and 106 controls.

**Sampling:** a line-list with 325 watery diarrhoea cases from the 28^th^ of September to 17 October 2020 was obtained at Mzilikazi clinic, and systematic random sampling was used to select cases with a sampling interval of 3 (325/106). Controls were randomly selected from the neighborhood of the cases. Controls were randomly selected at the 10^th^ house from where the enrolled case resided. If there were less than 10 houses from the house of the sampled control, the control was selected from the last house on the street.

**Data collection:** following the diarrhoea surveillance information from the clinic, health care workers at the clinic, and the city health department´s directorate were interviewed to obtain an overview on the outbreak. The surveillance data, line-list and clinical records were reviewed to confirm the outbreak. An interviewer-administered questionnaire and checklist was used to collect data. The questionnaire captured socio-demographic characteristics of respondents, signs and symptoms of illness, knowledge on diarrhoea, potential exposures and risk factors. A checklist adapted from the Integrated Disease Surveillance and Response (IDSR) technical guidelines was used to assess the department´s preparedness and response [8].

**Availability of data and materials:** the data sets generated and analyzed during the current study are available from the corresponding author on reasonable request.

### Data analysis

**Descriptive epidemiology:** the outbreak was described by person, place, and time. Microsoft Excel was used to analyze the line-listed cases by gender, age and common signs and symptoms. The software was also used to construct the epidemic curve. The Quantum Geographic Information System (QGIS) software was used to describe the spatial distribution of cases with reference to boreholes in the catchment area.

**Analytic epidemiology:** data were captured and analysed using Epi infoTM version 7. Statistical significance was set at 5%. Medians, proportions and frequencies were generated. The measure of normality used for age was the median. Wilcoxon rank-sum test was done to test for statistical difference for the median age for cases and controls. Odds ratios (OR) and 95% confidence intervals were generated and Chi-square test for significance was done at 5% significance level. For variables with more than two categories, one of the categories was assigned to be the reference category. Bivariate analysis to identify risk and protective factors were carried out, and stratified analysis was done to check for confounding and identify effect modification. Factors from the bivariate analysis with p-values = 0.25 were fitted into the regression model and backward logistic regression to determine factors independently associated with contracting diarrhoea was done. Adjusted Odds Ratios (aOR), 95% confidence intervals and p-values were generated.

**Environmental assessment:** a walk-through assessment on the availability and suitability of the water storage containers, and cleanliness of toilets at household level was done. Observation in the residential areas for the presence of burst sewer pipes, and waste dump sites was also done.

### Microbiological analysis

**Water:** a total of 11 water samples from boreholes and households were collected for microbiological and chemical analysis. The samples were tested using national standards for microbial and chemical test for water, by the Standards Association of Zimbabwe (SAZ), Standard 560: 1997.

**Stool:** thirteen stool samples were collected and sent for microscopy and culture at a medical laboratory. Stool samples were tested for watery diarrhoea causing coliforms

### Operational definitions

**Knowledge on diarrhoea:** score on the 3-point Likert scale, where a score of 0-4 was poor knowledge, 5-7 fair knowledge, and 8-10 good knowledge.

**Unsafe water for human consumption:** the total bacterial count was above 100 coliform forming units per millilitre, the total coliform count greater than 30 coliform forming units per 100 millilitres, and if there was presence of faecal coliforms.

**Drinking boiled water:** boiling and cooling water before drinking it.

### Permission and ethical considerations

Permission to conduct the study was obtained from the Bulawayo City Council (BCC) and the Health Studies Office. A written informed consent was obtained from each study participant. Confidentiality was assured to the respondents by not recording participant names on the questionnaires. The coronavirus disease infection prevention and control strategies were adhered to as the interviewer maintained physical distancing and face masks were properly donned during the interviews. Windows were kept open during the interviews and where practical, interviews were conducted outside the houses.

## Results

### Descriptive epidemiology

#### Person

A total of 486 cases of watery diarrhoea were attended to between the 28^th^ of September and the 9^th^ of December 2020. Of these, (275/486) 57% were females, and males contributed (211/486) 43%. All the cases were residents of the Mzilikazi clinic catchment area. Children under the age of five years contributed (165/486) 34%, while only (28/486) 6% were at least 65 years old for all the cases line listed. No deaths were reported. The three most common symptoms were diarrhoea (486/486) 100%, abdominal pains (159/486) 32.7% and vomiting (91/486), 19%. A total of (322/486) 66% cases who presented with watery diarrhoea were managed using an oral antibiotic, while (453/486) 93% were rehydrated.

#### Place

**Figure 1:**
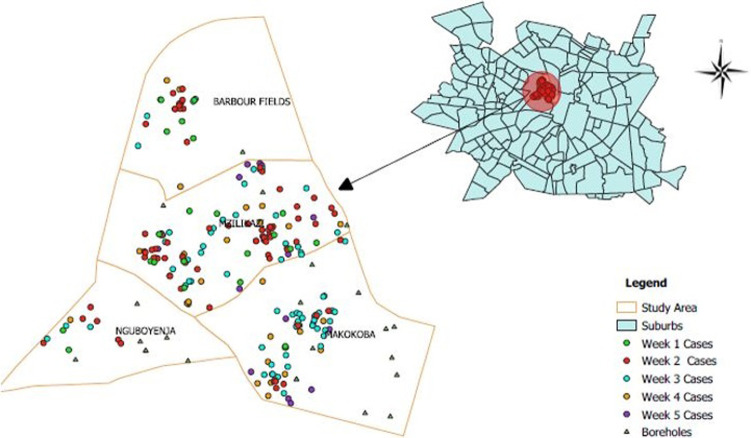
distribution of diarrhoea cases in the Mzilikazi clinic catchment area, Northern suburb district, Bulawayo, 2020

Figure 1 shows the spot map of cases mapped against boreholes in the Mzilikazi clinic catchment area. The spot map shows clustering of cases around boreholes particularly in Makokoba, and Mzilikazi townships.

#### Time

**Figure 2:**
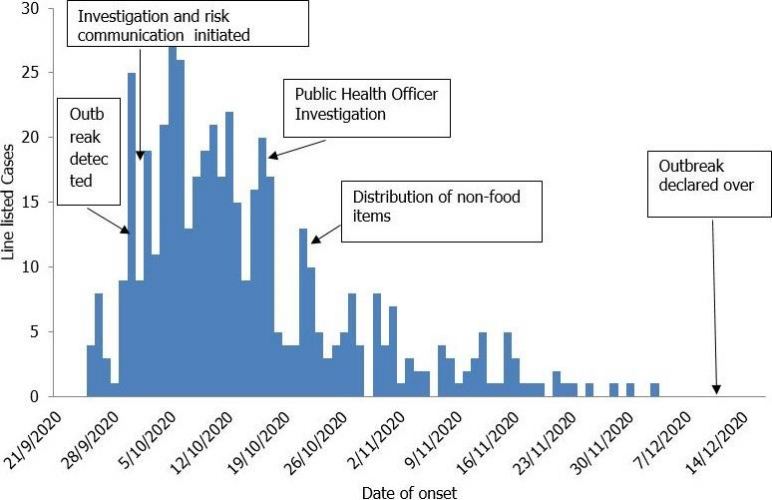
epidemic curve of watery diarrhoea outbreak in Mzilikazi clinic catchment area, Bulawayo, Zimbabwe, 2020

The epidemic curve suggested a mixed continuous common source and propagated mode of transmission. The Mzilikazi clinic catchment area watery diarrhoea outbreak was detected on the 30^th^ of September 2020, and a team was sent to investigate on the 1^st^ of October 2020. The epicurve shows multiple peaks with the highest number of cases per day (n=27) reported on the 5^th^ of October 2020 (Figure 2).

#### Environmental assessment

Twenty-nine percent (62/212) of the visited houses had dirty toilets and (72/212) 34% of the households visited were storing water for domestic use in dirty and open containers. Sewer blockages were also observed in the clinic’s catchment area. Residents of the Mzilikazi clinic catchment area were also observed accessing water from some decommissioned boreholes. These boreholes had been decommissioned by the BCC as the water had been deemed un-safe for human consumption. The Mzilikazi clinic catchment area was also observed to be densely populated.

### Microbiological analysis

#### Water

Five of the 11 water samples had results that indicated that the water was not fit for human consumption. Two were from boreholes while three were from water stored at household level.

#### Stool

A total of two of the 13 samples had growth of *Shigella flexneri* and one sample had growth of *Pseudomonas aeruginosa*.

#### Epidemic preparedness and response

The outbreak was detected early as the Bulawayo city health department was already on alert. The department had received events-based surveillance information from the community. The clinic had adequate resources for case management and was over-stocked with intravenous fluids.

### Analytic epidemiology

#### Demographic characteristics

We recruited 106 cases and 106 controls. The majority of the cases (62/106) 58.5% were females, and the majority of controls (59/106) 55.7% were males. The median age was 30 years (first quartile, Q1=12, third quartile, Q3=46), and 30 years (Q1= 22, Q3= 48) for cases, and controls respectively. There was no statistically significant difference in the medians for cases and controls (p-value = 0.14). The demographic characteristics of cases and controls were comparable (Table 1).

**Table 1:** demographic characteristics of cases and controls, Mzilikazi clinic catchment area watery diarrhoea outbreak, 2020

Variable	Cases n (%)	Controls n (%)	P-value
Gender			
Male	44 (41.5)	59 (55.7)	0.04
Female	62 (58.5)	47 (44.3)	
Age of Cases/Controls in years	Median =30 Q1=12; Q3=46	Median =30 Q1=22; Q3=48	0.14
Highest level of education attained			
	26 (24.5)	11 (10.4)	ref
None			
Primary	21 (19.8)	20 (18.9)	0.09
Secondary	52 (49)	63 (59.4)	<0.01
Tertiary	7 (6.7)	12 (11.3)	0.02
Religion			
None	3 (3)	4 (4)	ref
Muslim	0 (0)	2 (2)	0.26
Orthodox	45 (42)	41 (39)	0.63
Apostolic	32 (30)	30 (28)	0.66
Pentecost	26 (25)	29 (27)	0.83
Employment status			
Employed	34 (32.1)	46 (43.4)	0.09
Unemployed	72 (67.9)	60 (56.6)	

n frequency of cases or controls per category of a variable, % proportion of cases or controls per category of a variable, ref reference category, Q1 first quartile, Q3 third quartile, statistical significance considered at p-value<0.05

### Knowledge assessment

A total of (8/106) 7.5% of the cases and (12/106) 11.3% of the controls had poor knowledge on diarrhoea (Table 2). However, the knowledge difference was not statistically significant (p-value = 0.48).

**Table 2:** knowledge assessment of cases and controls, Mzilikazi clinic catchment area watery diarrhoea outbreak, 2020

Variable	Cases n (%)	Controls n (%)
What causes diarrhoea?	92 (86.8)	94 (88.7)
How is infection causing diarrhoea transmitted?		
Not practising hand washing	91 (85.4)	102 (96.2)
Drinking contaminated water	100 (94.3)	94 (88.7)
Consuming contaminated food	58 (54.7)	73 (68.9)
What do you do at home when someone contracts diarrhoea?		
Give Salt and Sugar Solution (SSS)	68 (64.2)	72 (67.9)
Visit clinic after having started person on SSS	18 (17)	34 (32.1)
What can we do at home to prevent diarrhoea?		
Domestic water treatment Practise hand washing	103 (97.2) 97 (91.5)	98 (92.5) 100 (94.3)
Store water in clean and closed containers	75 (70.8)	83 (78.3)
How is SSS prepared?	27 (25.7)	43 (41)

**n**: frequency of cases and controls who correctly responded to the question, **%:** proportion of cases and controls who correctly responded to the question

### Bivariate analysis of risk factors for diarrhoea

The statistically significant risk factors for contracting diarrhoea were being under-five years old [OR =3.49; 95% CI (1.42-8.62)], being female [OR =1.8; 95% CI (1.03-3.05)], eating cold foods in the past seven days [OR =1.94; 95% CI (1.08-3.50)], drinking borehole water [OR =2.73; 95% CI (1.56-4.75)] and storing water in an open container [OR =2.51; 95% CI (1.36-4.61)]. Boiling drinking water [OR =0.34; 95% CI (0.19-0.62)], and using soap for hand washing [OR =0.47; 95% CI (0.26-0.85)] were significant protective factors against contracting watery diarrhoea (Table 3).

**Table 3:** factors associated with contracting watery diarrhoea in the Mzilikazi clinic catchment area, Bulawayo, Zimbabwe 2020

Variable	Exposure Status	Cases n=106 (%)	Controls n=106 (%)	cOR (95% CI)	P-value	aOR (95% CI)	P-value
Age under-five years	Yes	21 (19.8)	7 (6.6)	3.49 (1.42-8.62)*	<0.01	5.73 (2.06-15.89)**	<0.01
	No	85 (80.2)	99 (93.4)				
Female	Yes	62 (58.5)	47 (44.3)	1.77 (1.03-3.05)*	0.04	1.72 (0.93-3.20	0.08
	No	44 (41.5)	59 (55.7)				
Eating cold foods in the last 7 days	Yes	41 (38.7)	26 (24.5)	1.94 (1.08-3.50)*	0.03	1.61 (0.81-3.22)	0.17
	No	65 (61.3)	80 (75.5)				
Drinking borehole water	Yes	68 (64.2)	42 (39.6)	2.73 (1.56-4.75)*	<0.01	2.66 (1.41-5.00)**	<0.01
	No	38 (35.8)	64 (60.4)				
Store water in open container	Yes	42 (39.6)	22 (20.8)	2.51 (1.36-4.61)*	<0.01	2.76 (1.38-5.53)**	0.01
	No	64 (60.4)	84 (79.2)				
Boiling drinking water	Yes	24 (22.6)	49 (46.2)	0.34 (0.19-0.62)*	<0.01	0.39 (0.20-0.75)**	<0.01
	No	82 (77.4)	57 (53.8)				
Use soap for hand-washing	Yes	64 (60.4)	81 (76.4)	0.47 (0.26-0.85)*	0.01	0.45 (0.23-0.88)**	0.02
	No	42 (39.6)	25 (23.6)				

*: significant variable in bivariate analysis, **: significant variable in multivariate analysis, cOR crude odds ratio, **aOR:** adjusted odds ratio, 95%CI 95% confidence interval. Statistical significance at p-value< 0.05

### Stratified analysis

The association between boiling drinking water and contracting diarrhoea stratified by sex were analyzed. The crude odds ratio for boiling drinking water always was within the stratum specific odds ratios. Therefore, the association between boiling drinking water and contracting diarrhoea was modified by sex (male or female). Females who boiled drinking water were 0.43 times [OR=0.43; 95% CI (0.20-0.97)] likely to contract diarrhoea compared to 0.25 times [OR=0.25; 95% CI (0.10-0.62)] among males who also boiled drinking water.

### Multivariate analysis of risk factors for diarrhoea

Drinking borehole water [aOR 2.66; 95% CI (1.41-5.00)], storing water in open container [aOR 2.76; 95% CI (1.38-5.53)] and being under the age of five [aOR 5.73; 95% CI (2.06-15.89)] were independent risk factors for contracting diarrhoea. Participants who drank borehole water were significantly 2.66 times more likely to have diarrhoea as compared to those who did not drink borehole water. Those who stored water in open containers were significantly 2.76 times more likely to have diarrhoea as compared to those who did not store water in open containers. Independent protective factors against contracting diarrhoea were drinking boiled water [aOR 0.39; 95% CI (0.20-0.75)] and using soap for hand washing [aOR 0.45; 95% CI (0.23-0.88)] Participants who drank boiled water had significant 61% reduced odds of having diarrhoea as compared to those who did not drink boiled water (Table 3).

## Discussion

The main findings from the study were that: *Shigella flexneri* was the causative agent, using borehole as an alternative source of water, storing water in open containers, and being under-five years old were risk factors while boiling drinking water and using soap for hand washing were protective factors against contracting watery diarrhoea.

The incubation period ranged from 16 hours to 4 days which is in congruent with the incubation period of *Shigella flexneri* isolated by the laboratory that ranges from 12 hours to 96 hours. An investigation of a *Shigella flexneri* outbreak by Saha *et al*. 2007 showed that the outbreak was caused by contamination of tap water and subsided following repair of the pipeline [9]. He *et al*. 2012 in China found that the diarrhoea outbreak they investigated was caused by drinking untreated water from a well contaminated with *Shigella flexneri* [10].

Zimbabwe has had two successive droughts in 2018 and 2019, and this affected the water levels at dams supplying most urban authorities with raw water. Bulawayo was not spared and was one of the most affected local authorities. Residents had to rely on alternative water sources like boreholes. Some of the borehole water samples tested were contaminated with coliforms, and residents were observed accessing water from decommissioned boreholes. The Mzilikazi clinic catchment area is densely populated and this puts a lot of pressure on sanitation infrastructure leading to frequent sewer shocks and blockages. The aging infrastructure, also contributed to the frequent sewer blockages. The sewer drainage capacity has thus been limited, with only 30% of sewer finding its way to the sewer treatment facilities, and 70% being discharged directly into streams and rivers. These factors may have led to the contamination of ground water [11].

Storing water in an open container was an independent risk factor for contracting watery diarrhoea. Bulawayo City Council introduced water shedding for 48 hours a week in 2019, which was then increased to 144 hours per week in 2020. The water rationing meant that residents had to store water in containers at their homes. Mzilikazi clinic catchment area is predominantly a high-density area, characterized by overcrowding. Most residents are unemployed, and a number of households depend on sub-letting in order to sustain their livelihoods [12]. Due to the difficult macro-economic environment in the country, most families could not afford to procure appropriate water storage containers. Contamination of water has been demonstrated to occur when water is poorly stored. Stored water samples collected from some of the households during the outbreak investigations, and tested were found to be contaminated with coliforms. Our study findings are consistent with Zorodzai *et al*. 2013 in Kadoma who found that using containers without a lid was a risk factor for contracting typhoid [13].

The diarrhoea outbreak mainly affected the under five-year age group. Children are more prone to gastroenteritis because their immune system is still developing, and also due to their poor personal hygiene [14]. Children spend most of their time outdoors playing, and in the Mzilikazi clinic catchment area where sewer was observed flowing in some areas, this could predispose the children to infections. Our findings are also consistent to a study by Maponga *et al*. 2011 where 82% of the people who presented with watery diarrhoea were children under five years of age [7].

One of the interventions designed to prevent diarrhoeal illnesses is home water treatment (HWT). HWT technologies in use include chlorination, filtration, solar disinfection, and boiling [15]. Drinking boiled water all the time was found to be a significant independent protective factor against contracting watery diarrhoea. This is biologically plausible as boiling water can kill some watery diarrhoea causing pathogens, including *Shigella flexneri* isolated [16]. Coliforms, including faecal coliforms were also detected from some of the water samples tested. The findings were consistent with Muti *et al.*, 2011 in Dzivaresekwa where drinking boiled water was found to be protective against typhoid [17]. The cost of energy particularly electricity and firewood in a predominantly low-income setting affected the uptake of the intervention. According to the Zimbabwe Vulnerability Assessment Committee (ZIMVAC) 2020 urban livelihoods assessment, the average monthly income for a family in high density areas like Mzilikazi was equivalent to 125 United States Dollars (USD), and was below the Total Consumption Poverty Line (TCPL) of USD 288 equivalent. The study also found that 7.4% of the families used firewood as a source of energy, and 6.6% of households with electricity in Bulawayo had electricity cut at least once in the six months preceding the survey [18,19]. Firewood which is enough to cook four meals costs one USD, which is a substantial amount if a family uses firewood only as the source for cooking energy. The cost of energy may therefore, present as a deterrent to the boiling of water for household use.

Water treatment using chlorine containing chemicals has been shown to be effective against diarrhoeal illness [15]. The study could not ascertain the benefits of using this intervention as it was not widely used. A study by Maponga *et al*. 2011, showed that using aqua tablets was protective against contracting watery diarrhoea (aOR= 0.44; 95%CI 0.26- 0.94) [7]. Following a typhoid outbreak in Harare, a study by Lantagne *et al*. 2011 recommended that as long as there is disruption or disturbance in the supply of centralized and treated municipal water, there is need to treat water from all sources daily. Social mobilization activities on HWT should therefore, be prioritized as part of the response [20].

Using soap for hand washing was also found to be an independent protective factor against contracting watery diarrhoea. This is also biologically plausible as soap has the ability to disinfect hands. Using soap for hand washing will therefore, interrupt the transmission of watery diarrhoea causing agents that are transmitted through the faeco-oral route. Financial constraints at household level could have affected the availability of soap for hand washing. A study conducted by Juru *et al*. 2018 following a Cholera outbreak in the City of Harare showed that (98/156) 63% of the households did not have soap for hand washing [21].

### Study strengths and limitations

The study strengths were that, it was inexpensive to conduct and results were obtained in a timely manner to aid the outbreak response. The Limitations of this study were that at the time of the study some of the cases had already recovered from the illness, and this could possibly introduce recall bias. Some of the study questions had a potential to introduce social desirability bias. We could not assess the use of chlorine-based HWT in the Mzilikazi clinic catchment area. To minimize recall bias, a well-structured data collection tool was used for both cases and controls, and the study participants were given enough time to think through before responding to the questions. To minimize social desirability bias, participants were only provided with a brief overview of the study to avoid priming them to respond in a socially desirable manner.

## Conclusion

The risk factors for contracting diarrhoea in the Mzilikazi clinic catchment area were: drinking borehole water, storing water in open containers and being under-five years old. Drinking boiled water was protective against diarrhoea and HWT using chlorine was not widely utilized. The cost of energy may present as a barrier for HWT through boiling. The findings suggest that under-ground water in the clinic’s catchment area may be contaminated rendering it unsafe for human consumption. With the old water and sewer reticulation infrastructure in highly populated urban settings, assuming that all ground water is contaminated may be life-saving. Based on our findings, we recommended social mobilization of the community on HWT including the use of chlorine-based water treatment chemicals, regular borehole water sample testing to guide targeted intervention, and to mobilize and distribute chlorine based water treatment chemicals, and appropriate water storage containers whenever there is severe water rationing in the city or during a diarrhoeal outbreak. Additionally for under-five year olds, care givers of these children should be educated on good hygiene practices at home, including ensuring that playing environments for the children are clean. Public health actions taken: based on the evidence from this study distribution of non-food items like soap, chlorine-based water treatment chemicals, and water storage containers was intensified. We intensified health education of the community on the importance of HWT through boiling or using chlorine-based water treatment chemicals particularly for borehole water and 681 households were reached. The Makokoba COVID-19 community taskforce was sensitized on the watery diarrhoea outbreak, and the preventive measures. The results of the study were presented to the Bulawayo city health department, and the urban councils of Zimbabwe health officers’ forum meeting.

### What is known about this topic

- *Watery diarrhoea outbreaks are caused by drinking contaminated water;*
- *Poor water storage is known to cause watery diarrhoea outbreaks;*
- *Diarrhoea outbreaks have been demonstrated to occur frequently in overcrowded urban settlements*.

### What this study adds

- *The study highlighted the low utilization of chlorine based HWT which has been demonstrated in other settings but had not been demonstrated in Bulawayo city, Zimbabwe*.
